# Social Incentives to Improve Mobility Among Elderly Patients After Radical Cystectomy

**DOI:** 10.1093/geroni/igaa057.2977

**Published:** 2020-12-16

**Authors:** Daniel Lee, Kenneth Covinsky, S Ryan Greysen

**Affiliations:** 1 University of Pennsylvania Health System, Philadelphia, Pennsylvania, United States; 2 University of California San Francisco, San Francisco, California, United States; 3 University of Pennsylvania, Philadelphia, Pennsylvania, United States

## Abstract

Radical cystectomy is a complex surgery that disproportionately affects elderly patients and is associated with high morbidity and mortality rates. We proposed a randomized control trial to improve mobility using gamification and social incentives while hospitalized, and immediately after surgery at home. Social incentives may be particularly useful in this population as the majority of cystectomy patients rely on a primary caregiver. There have been multiple challenges in this interdisciplinary work to utilize innovative technologies. This would represent one of the first use of gamification and behavioral economic principles in postoperative patients, so there are questions about how patients would respond to gamification while recovering postoperatively. There were challenges in patient selection and interdisciplinary collaboration to consider, especially in an elderly population that may not have ready mobile access. By collaborating across disciplines, we hope to make significant improvements in postoperative outcomes for elderly patients by utilizing innovative interventions.

